# Effect of Land Use Conversion on Surface Soil Heavy Metal Contamination in a Typical Karst Plateau Lakeshore Wetland of Southwest China

**DOI:** 10.3390/ijerph17010084

**Published:** 2019-12-20

**Authors:** Caili Sun, Sixi Zhu, Bin Zhao, Wujiang Li, Xiaoye Gao, Xiaodan Wang

**Affiliations:** 1 College of Eco-Environmental Engineering, Guizhou Minzu University, Guiyang 550025, China; suncaili2007@126.com (C.S.); zhaobin20188@163.com (B.Z.); 18798757657@163.com (W.L.); gaoxiaoye1220@163.com (X.G.); wangxiaodan2007@163.com (X.W.); 2 The Institute of Karst Westland Ecology, Guizhou Minzu University, Guiyang 550025, China

**Keywords:** land uses conversion, heavy metal contamination, ecological risk assessment, karst plateau, lakeshore wetland

## Abstract

Land use conversion could directly or indirectly influence heavy metal geochemistry by changing soil properties. The aim of this study was to explore the effect of land use conversion on surface soil heavy metal contamination in the karst plateau lakeshore wetlands of Southwest China. Based on this, a total of 120 soil samples were collected from 30 sites from different types of land uses (farmlands, grasslands and woodlands) around a lake in Suohuangcang National Wetland Park in August 2017. Contents of As, Cd, Cu, Cr, Hg, Pb and Zn were analyzed, and soil heavy metal contamination was assessed in all three land use types. Results showed that land use transformation from farmland to grassland or woodland was not conducive to the release of soil heavy metal. Surface soil of all three land use types have been moderately polluted by As, Cr, Pb, and Zn, and grassland and woodland also had moderate Cd contamination. The pollution load index (PLI) results revealed low heavy metal contamination in grassland and woodland but no contamination in farmland. Although the integrated contamination in the studied region did not pose a serious potential ecological risk (RI < 150), it might affect human health through the water supply and food chain. Therefore, it is necessary to monitor and control As, Cd, Cr, Pb, and Zn concentrations of surface soil through controlling pollutants, improving waste treatment, as well as strengthening supervision and management in the vicinity of the Suohuangcang National Wetland Park.

## 1. Introduction

Wetlands play an important role in providing vital ecosystem services to populations living in their vicinity, such as agricultural production, water quality maintenance and recreation [1,2]. However, most wetlands have been reclaimed or occupied with economic and social activities, especially in developing countries [3], and Gong et al. (2010) reported that approximately 60,404 km^2^ of natural inland wetlands in China disappeared between 1990 and 2000 because of the population boom and subsequent human disturbances [4]. Soil contamination coming from anthropogenic activities, such as agricultural practices, industrial processes, and urban activities including domestic waste and vehicles emission, seriously influence and hinder the realization of their ecosystem service [5]. Heavy metals from wetland soils can be released to adjacent waters through agricultural runoff [3,6] and potentially affect terrestrial and aquatic communities. For lakeshore wetlands, these effects are more pronounced, since the water in a lake is stagnant, allowing heavy metals to easily accumulate.

Recently, heavy metal contamination in soil has gained wide attention for its toxicity, persistency, and non-biodegradability in the environment and threat to plant, animals and humans through water and food chain transport [7]. Land use conversion has been demonstrated to directly or indirectly influence the geochemical position of heavy metals through changing the properties of the soil [8,9]. Many studies indicated that farmlands usually exhibited significantly higher heavy metal contents compared to other land use types due to the continuous application of agrochemicals, which contain a variety of heavy metals as impurities [9,10,11]. However, is this true for all cases? No, Bai et al. (2010) found low heavy contamination levels for cultivated soils relative to abandoned tilled soil in wetland soils along a typical plateau lake of China [3]. The difference in research results was mainly because land use conversion has important consequences for soil physical, chemical and biological processes, which can indirectly affect heavy metal geochemistry [9], and thus deeper analysis was needed under specific conditions.

The southwest karst region in China, centered in Guizhou Province, is the largest continuous karst region in the world [12], and is characterized by fragile ecological environments and strong soil erosion [13]. Land use conversion in this region was part of the state-funded “Grain for Green Project”, which was launched by the Chinese government in 1999 to decrease erosion by converting farmlands to forest and grasslands [14]. Previous research on the response of soil quality to land use patterns mainly focused on the variation of nutrient parameters [15,16]. The effects of land use conversion on heavy metal accumulation in soil have rarely been investigated, especially in wetlands.

As discussed above, land use conversion can affect heavy metal geochemistry [8], but what is the specific effect considering the particular soil properties of lakeshore wetland? We hypothesized that heavy metal contamination in surface soil could be reduced after land use conversion from farmland to grassland or woodland. Based on this, seven heavy metals in surface soil of three land use types (farmlands, grasslands and woodlands) around a lake were investigated in Suohuangcang National Wetland Park, and the primary objectives of this study were (1) to analyze changes in heavy metals after land use conversion; and (2) to assess heavy metal contamination levels in these three land use types. The results of this study will provide guidelines for soil environmental protection and can also facilitate regional environmental risk assessment.

## 2. Materials and Methods

### 2.1. Site Description

Suohuangcang National Wetland Park is located in northwestern Guizhou (26°53′0″–26°54′40″N, 104°11′40″−104°12′40″E), 3.5 km away from Weining County and 2.5 km away from Caohai National Nature Reserve, covering an area of 244.67 hm^2^. As a karst lake wetland system in Yun-Gui Plateau, it is a valuable state reserve in China for migrant birds, such as Black-necked Crane (*Grus nigricollis*), White Stork (*Ciconia ciconia*), and other rare birds. It has a temperate climate with an average temperature of 10.5 °C and a mean annual rainfall of approximately 900 mm. The average relative humidity is 79%, with distinct wet and dry seasons, and the wet season with higher humidity lasts from May to October [17]. In Suohuangcang National Wetland Park, carbonate rocks are widespread, and surface soil is dominated by calcareous soil [18].

Suohuangcang National Wetland Park has a long agricultural history due to its large population. As a result, land resources have been critically depleted by soil erosion, and the eco-environment has been polluted by multiple contaminants. In 1999, the Chinese government launched “Grain for Green Project”, and many farmlands were abandoned or converted to grasslands or woodlands [14]. Nowadays, farmlands, grasslands and woodlands are the most common land use types and widely distributed near lake shorelines. The main crops cultivated on farmlands are vegetables, corn and potatoes. The dominant herbaceous plants grown in grasslands are *Conyza canadensis*, *Juncus effuses* and *Trifolium repens*. Moreover, *Pinus yunnanensis* and *Pinus armandii* are common tree species in woodlands.

### 2.2. Soil Sampling and Analysis

In this study, surface soil samples (0–20 cm) were collected from 30 sites around the lake in August 2017. Every site included 4 sampling points, and each point was sampled three times to obtain a composite soil sample. In total, 120 composite soil samples composed of 40 farmland soils, 44 grassland soils and 36 woodland soils were collected. Soil samples were placed in polyethylene bags and brought to the laboratory, where they were air-dried at room temperature and sieved through a 2 mm nylon sieve to remove coarse debris. When completely dry, all soils were ground with a pestle and mortar in order to pass a 0.15 mm nylon sieve.

**Figure 1:**
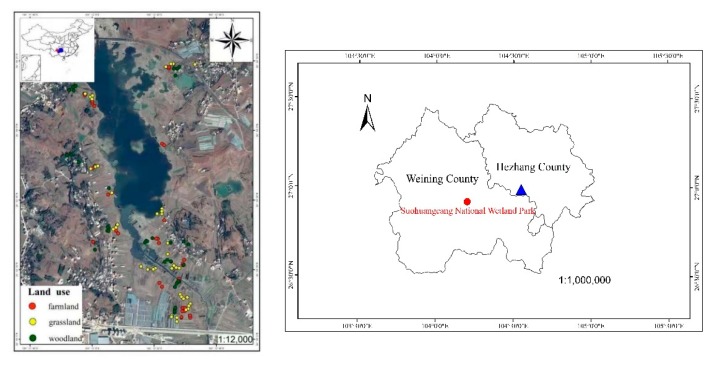
Study area and soil sampling points. Note: Blue area and the red dot in the map of China respectively represent Guizhou province and Suohuangcang National Wetland Park. The blue triangle in Hezhang county boundary indicates main area of zinc smelting using an indigenous method.

The powdered soil sample (0.3 g) was accurately weighted into a 50 mL Teflon crucible, and was initially digested with 5 mL concentrated HCl. It was then digested with a mixture acid system of “HNO_3_-HF-HClO_4_” (5, 4, and 2 mL, respectively). Finally, the digested solution was diluted to 25 mL with deionized water [19]. The contents of Cd, Cu, Cr, Pb and Zn were determined using an atomic absorption spectrophotometer (FAAS, Perkin-Elmer, Norwalk, OH, USA). As and Hg content was determined by Atomic Fluorescence Spectrometry (AFS, Titian, China). Reagent blanks, triplicates and standard reference materials (GBW07403, Chinese Academy of Measurement Sciences) were used for quality assurance/quality control. The recovery percentages ranged from 91.8% to 102.2%, indicating a good agreement between the measured and the certified values. (Figure 1)

### 2.3. Assessment of Soil Contamination

#### 2.3.1. Assessment of Heavy Metal Contamination

The overall pollution status of single heavy metal was assessed by the contamination factor (CF), which was defined as follows [20]:

(1) $$\mathrm{CF}=\frac{{Me}_{sample}}{{Me}_{baseline}}$$

where ${Me}_{sample}$ is the measured concentration of heavy metal, and ${Me}_{baseline}$ is the natural abundance of a given heavy metal. Values of the CF are characterized as: low degree (CF < 1), moderate degree (1 ≤ CF < 3), considerable degree (3 ≤ CF < 6), and very high degree (CF ≥ 6).

However, as heavy metals always occur in soils as complex mixtures with great variation, the pollution load index (PLI) was used to determine and compare the integrated pollution status of combined contaminants at sampling sites [21]. The PLI was calculated by the following equation:

(2) $${\mathrm{PLI}=(CF}_{1}{\times CF}_{2}{\times CF}_{3}{\cdots \times CF}_{n}{)}^{1/n}$$

where ${CF}_{1}$ is the CF value of metal *n*. Value of PLI < 1 indicates no contamination in the sampling site, and PLI > 1 means that the sampling site has been subjected to contamination.

#### 2.3.2. Assessment of the Potential Ecological Risk

The potential ecological risk of heavy metals was evaluated by the potential ecological risk index (RI), which was proposed by [20], and defined as follows:

(3) $$E_{r}^{i}{=T}_{r}^{i}\times \mathrm{CF}$$

(4) $$\mathrm{RI}=\overset{n}{\underset{i=1}{\sum }}E_{r}^{i}$$

where CF is the contamination factor, $T_{r}^{i}$ is the response coefficient for the toxicity of the single heavy metal. According to the Hakanson method, the corresponding coefficients based on its toxicity were: Hg = 40, Cd = 30, As = 10, Cu = Pb = 5, Cr = 2, and Zn = 1 [20]. $E_{r}^{i}$ is the potential ecological risk index of an individual element. Grading standards of the potential ecological risk of heavy metals are shown in Table 1.

### 2.4. Statistical Analysis

Descriptive statistics including the average, maximum, minimum, standard deviation and coefficient of variation were performed after analysis. Differences in heavy mental contents among land use types were assessed by a one-way analysis of variance and multifractal comparison. Site-average values were compared by LSD tests at *p* < 0.05. Pearson correlation analysis and principal components analysis (PCA) based on standardized site-average values were carried out to identify the potential sources of heavy metals. All statistical analyses and plots were performed using the vegan and ggplot 2 packages in R v.3.6.0 [22].

## 3. Results

### 3.1. Heavy Metal Contents in Surface Soil of Different Land Use Types

Statistical results for the contents of six heavy metals in farmland, grassland and woodland surface soil are shown in Table 2. Compared with background values of uncontaminated natural soil in Guizhou province, the contents of Cr, Zn, As and Pb in three land use types and the content of Cd in grassland and woodland were higher, but the contents of Cu and Hg were lower. Furthermore, most heavy metal contents exhibited large degrees of variation except for Cd in grassland and Hg in all land use types, with a variable coefficient larger than 15%.

**Figure 2:**
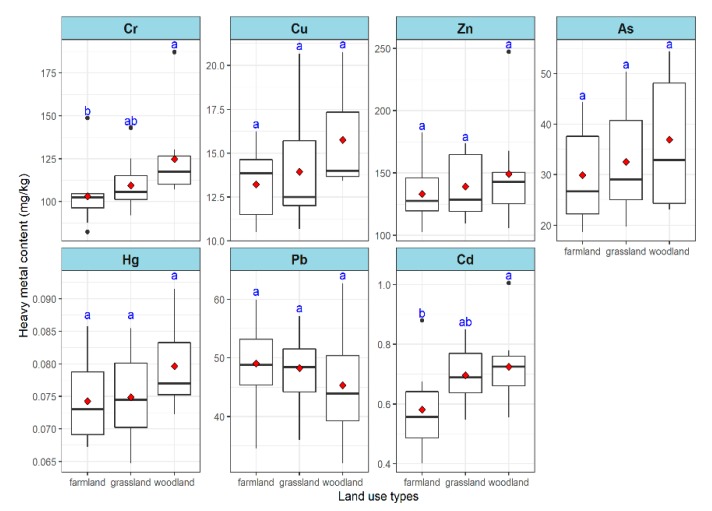
Contents of heavy metal in surface soil of different land use types. Note: different letters above the bar plot indicate significant differences at *p* < 0.05. Post-test results of multiple comparison are shown in the Supplementary File (Table S1).

As shown in Figure 2, the average contents of Cr and Cd (124.824 and 0.725 mg/kg, respectively) in woodlands were significantly higher than that in farmlands. However, no significant difference of Cu, Zn, As, Hg and Pb contents were observed among land use types.

### 3.2. Assessment of Ecological Risks in Different Land Use Types

According to the definition of CF and PLI, surface soils of all land use types were moderately contaminated by Cr, Zn, As and Pb, with CF between 1 and 2, and were not contaminated by Cu and Hg, with CF lower than 1. Further, in surface soil of grassland and woodland, there was moderate Cd contamination, with CF of 1.057 and 1.100, respectively. The PLI values of surface soil in farmlands, grasslands, and woodlands were 0.948, 1.005, and 1.078, respectively (Table 3).

Ecological risk assessment results showed that surface soil of farmlands, grasslands and woodlands were categorized as low pollution level, as *E^i^_r_* and *RI* values were lower than 40 and 150, respectively (Table 4).

### 3.3. Source Identification of Heavy Metals in Different Land Use Types

Pearson correlation analysis was used to investigate the relationships between different heavy metals and support the results obtained by PCA (Table 5 and Table S2). The results indicated that Cd, Cr, Cu and Zn were highly positively correlated with each other for all three land use types, except that there were no correlations between Cr and Cu in grassland and woodland and between Cr and Cd in grassland. Further, As was positively correlated with Cu for all kinds of land use types.

**Figure 3:**
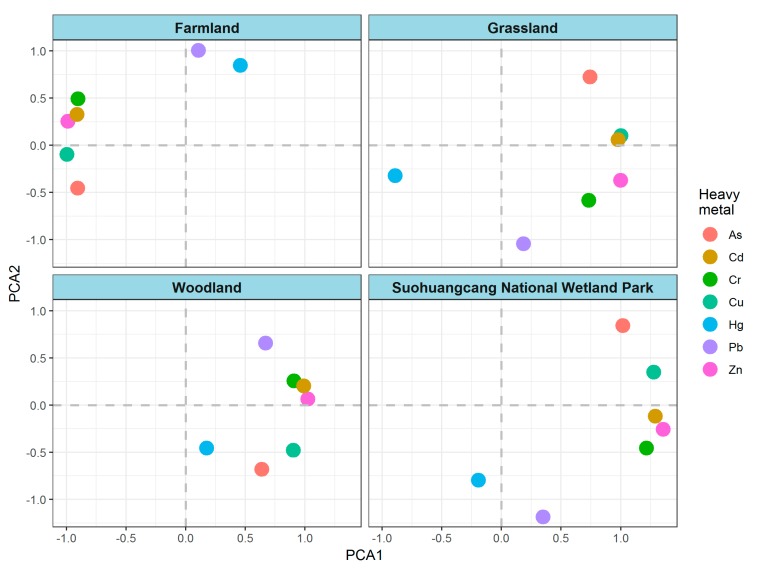
Principal component analysis (PCA) results of six heavy metals in surface soil of different land use types.

PCA was used to determine the sources of six heavy metals in surface soil of different land use types (Figure 3). For farmlands, grasslands and woodlands, the first two principal components represented 88.50% (PCA1 58.74%; PCA2 29.76%), 84.60% (PCA1 58.24%; PCA2 26.36%) and 80.14% (PCA1 60.85%; PCA2 19.29%) of the total variance, respectively. For Suohuangcang National Wetland Park, the first two principal components represented 76.86% (PCA1 54.73%; PCA2 22.13%) of the total variance. According to the load weight on two principal components, six heavy metals were generally divided into three groups—group 1 (Cd, Cr, Cu and Zn), group 2 (As) and group 3 (Pb and Hg)—no regardless of the land use type in Suohuangcang National Wetland Park. Specifically, Cd, Cr, Cu and Zn greatly contributed to PCA1, and Pb was closely associated with PCA2.

## 4. Discussion

### 4.1. Effect of Land Use Types on Heavy Metal Concentration in Surface Soil

Surface soil of grasslands and woodlands in Suohuangcang National Wetland Park was mainly contaminated by As, Cd, Cr, Zn and Pb, and farmland was contaminated by As, Cr, Zn and Pb, since higher concentrations of these heavy metals were observed compared to background values in soils of GuiZhou province. In general, external sources of heavy metals accessing soil were mainly from natural weathering and anthropogenic activities [24,25]. In this study, the geological context was similar. So, excessive amounts of Cd, Cr, Zn, As and Pb were mainly related to human activities. Historically, local zinc smelting using an indigenous method was common in Hezhang county, not far from the study site, which was considered the primary source of Zn and Cd contamination (Figure 1), and Hadzi et al. (2019) also found close correlation between Cd and Zn in surface soil of mining areas [26]. Contamination of Cr, As and Pb is likely due to artificial coal burning and industrial activities. Overall, anthropogenic practices are the main source of heavy metal contamination in surface soils in Suohuangcang National Wetland Park.

Changes in heavy metal concentration in the surface soil under different land use types indicated that the transformation of land use types from farmlands to grasslands and woodlands was not beneficial to the removal of heavy metals, as no significant difference of Cu, Zn, As, Hg and Pb contents was observed among farmland, grassland and woodland and only a significantly higher content of Cr and Cd was found in woodlands compared to farmland. Similar results were reported by Bai et al. (2010), showing that soil heavy metals of As, Cd, Cu, Pb and Zn decreased after cultivation, but increased after the abandonment of cultivated wetlands in the Yunnan province of China [3]. However, many studies also showed that farmland cultivation led to increased heavy metals contamination in soil due to fertilizers and pesticides addition [27,28]. The reason for these variances is due to the difference in farmland soil properties and cultivation practices that alter the geochemical behavior of heavy metals in soil [9]. In this study, low heavy metal contamination in grassland and woodland mainly came from anthropogenic practices, containing point source or non-point source pollution, which discharged heavy metals like Cd, Cr, Zn, As and Pb into the environment [17,29]. However, no contamination in farmland was mainly because of their exclusive location (within land-lake ecozone), where superior conditions could offset some emissions of heavy metal. But, in general, heavy metal pollution in Suohuangcang National Wetland Park was not serious.

### 4.2. Response of Ecological Risk in Surface Soil to Land Use Types

Corresponding to statistics results of heavy metal contents, CF values indicated that all three land use types were moderately contaminated with Cr, Zn, As and Pb, and grassland and woodland also showed moderate Cd contamination. PLI values in farmlands, grasslands and woodlands were 0.948, 1.005 and 1.078 respectively, which suggested that farmland is less polluted than grassland, which in turn is less polluted than woodland. This result was inconsistent with the previous hypothesis, but it was reasonable for the following reasons. Firstly, the increase in Cr and Cd content in woodland could be related to soil organic matter (SOM) [18,30] that can act as a sink for heavy metals through absorbing and retaining heavy metals in soils [31,32]. Land use transformation from farmlands to woodlands is usually beneficial for the improvement of soil SOM content [33,34], and thus the highest concentration of Cr and Cd exhibited in woodlands is most likely attributed to the improvement of soil SOM compared to farmlands. Secondly, soil moisture was another reason explaining why Cr and Cd contents in woodland were significantly higher than that in farmland. In the current study, farmlands were located at a land-lake ecozone, and were closer to the lake than the woodlands, showing abundant soil moisture. Soil moisture could greatly affect the solubility, toxicity, bioavailability, and mobility of Cd and cause it redistribution in soils [35]. Thirdly, the study site is typical karst wetland characterized by large soil porosity and even fissures, which provide a route for water movement and infiltration [3,13], leading to heavy metal leaching [36]. Finally, plant uptake and root distribution also contributed to the relatively higher Cr and Cd content value in woodland [30]. Vegetables or crops in farmlands were harvested after they were ripe and roots of plants in farmlands were mainly concentrated in the surface soil layer in contrast to woodlands. Heavy metals content in surface soil was thus reduced though the removal of plant materials that contained considerable heavy metal levels through root uptake.

The potential ecological risk assessment showed low contamination levels for all heavy metals in farmlands, grasslands and woodlands. However, although this study did not show a serious potential ecological risk from the integrated heavy metal concentration, it is still necessary to keep monitoring and controlling heavy metal pollution in Suohuangcang National Wetland Park, since it could affect the quality of agricultural products and impact human health through the water supply and food chain [10].

### 4.3. Source Analysis of Heavy Metals in Different Land Use Types

The PCA results suggested that the source of Cd, Cr, Cu and Zn was different from that of Pb, for all three land use types, since Cd, Cr, Cu and Zn greatly contributed to PCA1 and Pb was closely associated with PCA2. Our findings were in accordance with those of Hu et al. (2017), where seven heavy metals were divided into three groups: group1 with Hg, Cd and Pb, group2 with As, and group3 with Cr, Cu and Zn [17]. It is well documented that Cd, Cr, Cu and Zn are usually present in metal smelting and electroplating [37]. Coincidentally, some vehicle repair plants and a zinc smelt area are also located not far from the research site (Figure 1) [17,38]. Since As showed a positive correlation with Cu, it may have a similar source input. Therefore, the source of As, Cd Cr, Cu and Zn might originate from zinc mining and industrial activities. Coal burning is still the main source of heat for cooking and warming for local residents due to a relatively outdated energy structure, which is considered to be mainly responsible for the increase in Pb contamination [22]. In addition, vehicular traffic and wastes from commodities also contribute to Pb pollution in soils [39,40]. Taken together, anthropogenic pollution greatly contributed to the high heavy metal contamination in surface soil. Therefore, controlling these heavy metals in the studied area can be achieved through strengthening supervision and management on the major source of pollution as well as improving waste treatment in the vicinity of the Suohuangcang National Wetland Park.

## 5. Conclusions

Surface soil of grasslands and woodlands in Suohuangcang National Wetland Park was mainly contaminated by As, Cd, Cr, Zn and Pb, and farmland was contaminated by As, Cr, Zn and Pb. Land use transformation from farmlands to grasslands or woodlands was not conducive to the release of heavy metals in Karst plateau wetland soil. The PLI results revealed that farmland was less polluted than grassland, which in turn was less polluted than woodland. The excess As, Cd, Cr, Zn and Pb contributed greatly to the increased PLI values in grassland and woodland, and they mainly came from anthropogenic pollution, such as mining activities, coal burning, and industrial emission. In general, the integrated contamination did not pose a serious potential ecological risk (*RI* < 95), but it is still necessary to monitor and control As, Cd, Cr, Zn and Pb concentrations in surface soil, as these heavy metals can affect human health through the water supply and food chain. This can be achieved through controlling pollutants, improving waste treatment, as well as strengthening the supervision and management in the vicinity of the Suohuangcang National Wetland Park.

## Figures and Tables

**Table 1:** Relationship between the potential ecological risk index of an individual element $E_{r}^{i}$ and pollution levels.

$E_{r}^{i}$	Ecological Risk Level of Single-Factor Pollution	RI	General Level of Potential Ecological Risk
$E_{r}^{i}<40$	low	RI < 150	low
$40\leq E_{r}^{i}<80$	moderate	150 ≤ RI < 300	moderate
$80\leq E_{r}^{i}<160$	considerable	300 ≤ RI <600	considerable
$160\leq E_{r}^{i}<320$	high	RI ≥ 600	very high
$E_{r}^{i}\geq$320	very high		

**Table 2:** Statistics results of heavy metal contents in surface soil of different land uses types.

Land Use Types	Heavy Metals	Sample Size	Heavy Metal Contents (mg/kg)			Standard Deviation	Variable Coefficient (%)	Background Value (mg/kg) [23]
			Mean	Min	Max			
farmland	Cr	40	103.175	70.667	173.667	19.857	19.246	95.900
	Cu	40	13.217	9.333	20.000	2.627	19.880	32.000
	Zn	40	133.158	90.333	205.333	27.622	20.744	99.500
	As	40	29.911	17.733	51.300	10.118	33.828	20.000
	Hg	40	0.074	0.061	0.095	0.009	12.176	0.110
	Pb	40	49.081	19.833	73.000	8.786	17.900	35.200
	Cd	40	0.581	0.402	0.88	0.136	23.355	0.659
grassland	Cr	44	109.371	74.333	171.000	17.916	16.381	95.500
	Cu	44	13.932	10.000	26.667	3.502	25.139	32.000
	Zn	44	139.197	100.667	205.667	26.589	19.101	99.500
	As	44	32.573	18.467	68.733	11.913	36.573	20.000
	Hg	44	0.075	0.055	0.105	0.011	14.674	0.110
	Pb	44	48.239	15.733	62.567	7.522	15.593	35.200
	Cd	44	0.697	0.547	0.851	0.103	14.816	0.659
woodland	Cr	36	124.824	74.667	206.333	32.552	26.078	95.500
	Cu	36	15.759	10.667	22.333	3.175	20.146	32.000
	Zn	36	149.176	92.667	272.667	47.164	31.616	99.500
	As	36	36.915	13.033	79.667	15.046	40.758	20.000
	Hg	36	0.080	0.066	0.100	0.010	12.315	0.110
	Pb	36	45.337	24.133	68.667	11.779	25.982	35.200
	Cd	36	0.725	0.555	1.006	0.127	17.493	0.659

**Table 3:** Concentration factor (CF) and pollution load index (PLI) of surface soil in different land use types.

Land Use Types	CF							PLI
	Cr	Cu	Zn	As	Hg	Pb	Cd	
farmland	1.076	0.413	1.338	1.496	0.675	1.394	0.882	0.948
grassland	1.140	0.435	1.399	1.629	0.681	1.370	1.057	1.005
woodland	1.302	0.492	1.499	1.846	0.724	1.288	1.100	1.078

**Table 4:** The potential ecological risk factors (*E^i^_r_*) and the potential ecological risk index (*RI*) of surface soil in different land use types.

Land Use Types	*E^i^_r_*							*RI*
	Cr	Cu	Zn	As	Hg	Pb	Cd	
farmland	2.152	2.065	1.338	14.955	27.009	6.972	26.446	61.463
grassland	2.281	2.177	1.399	16.287	27.223	6.852	31.712	63.071
woodland	2.603	2.462	1.499	18.457	28.960	6.440	32.995	66.862

**Table 5:** The correlations of heavy metals in surface soil of different land use types.

Land Use Types	Heavy Metals	Cr	Cu	Zn	As	Hg	Pb	Cd
farmland	Cr	1						
	Cu	0.776 **	1					
	Zn	0.864 **	0.860 **	1				
	As	0.481	0.814 **	0.662 *	1			
	Hg	−0.035	−0.463	−0.194	−0.62	1		
	Pb	0.357	−0.154	0.118	−0.498	0.695 *	1	
	Cd	0.847 **	0.641 *	0.835 **	0.634 *	−0.116	0.169	1
grassland	Cr	1						
	Cu	0.484	1					
	Zn	0.742 **	0.775 **	1				
	As	0.166	0.642 *	0.416	1			
	Hg	−0.423	−0.739 **	−0.587	−0.691 *	1		
	Pb	0.526	0.093	0.48	−0.518	0.116	1	
	Cd	0.434	0.821**	0.849**	0.593	−0.634 *	0.098	1
woodland	Cr	1						
	Cu	0.632	1					
	Zn	0.893 **	0.835 **	1				
	As	0.284	0.879 **	0.556	1			
	Hg	0.116	0.26	0.122	0.069	1		
	Pb	0.606	0.278	0.650	0.021	−0.073	1	
	Cd	0.879 **	0.722 *	0.976 **	0.439	0.110	0.719 *	1

Note: ** correlation is significant at the 0.01 level (2-tailed); * correlation is significant at the 0.05 level (2-tailed). The correlation test results are shown in the Supplementary File (Table S2).

## Supplementary Materials

The following are available online at https://www.mdpi.com/1660-4601/17/1/84/s1, Table S1: Post-test results of multiple comparison for heavy metal content in different land use types, Table S2: Correlations analysis of heavy metals in surface soil of different land use types.
