# Supplementary material for: Effect of Land Use Conversion on Surface Soil Heavy Metal Contamination in a Typical Karst Plateau Lakeshore Wetland of Southwest China
Source: Int J Environ Res Public Health. 2019 Dec 20;17(1):84. doi: 10.3390/ijerph17010084 (PMC6982003; doi:10.3390/ijerph17010084)
Supplement: Supplementary file 1 [file ijerph-17-00084-s001.pdf]

Table S1. Post-test results of multiple comparison for heavy metal content in different land use types.

Heavy metal	Land use types	Land use types	Significance test (*P* value)
Cr	farmland	grassland	0.467
		woodland	0.021
	grassland	farmland	0.467
		woodland	0.085
	woodland	farmland	0.021
		grassland	0.085
Cu	farmland	grassland	0.549
		woodland	0.050
	grassland	farmland	0.549
		woodland	0.144
	woodland	farmland	0.050
		grassland	0.144
Zn	farmland	grassland	0.656
		woodland	0.266
	grassland	farmland	0.656
		woodland	0.476
	woodland	farmland	0.266
		grassland	0.476
As	farmland	grassland	0.59
		woodland	0.184
	grassland	farmland	0.590
		woodland	0.395
	woodland	farmland	0.184
		grassland	0.395
Hg	farmland	grassland	0.844
		woodland	0.096
	grassland	farmland	0.844
		woodland	0.128
	woodland	farmland	0.096
		grassland	0.128
Pb	farmland	grassland	0.807
		woodland	0.306
	grassland	farmland	0.807
		woodland	0.416
	woodland	farmland	0.306
		grassland	0.416
Cd	farmland	grassland	0.039
		woodland	0.016
	grassland	farmland	0.039
		woodland	0.611
	woodland	farmland	0.016
		grassland	0.611

Table S2. Correlations analysis of heavy metals in surface soil of different land use types.

	Correlation test	Cr	Cu	Zn	As	Hg	Pb	Cd
Cr	Correlation coefficient (Pearson)	1						
	Significance test-*P* value (bilateral)							
Cu	Correlation coefficient (Pearson)	0.776**	1					
	Significance test-*P* value (bilateral)	0.008						
Zn	Correlation coefficient (Pearson)	0.864**	0.860**	1				
	Significance test-*P* value (bilateral)	0.001	0.001					
As	Correlation coefficient (Pearson)	0.481	0.814**	0.662*	1			
	Significance test-*P* value (bilateral)	0.159	0.004	0.037				
Hg	Correlation coefficient (Pearson)	−0.035	−0.463	−0.194	−0.62	1		
	Significance test-*P* value (bilateral)	0.923	0.178	0.591	0.056			
Pb	Correlation coefficient (Pearson)	0.357	−0.154	0.118	−0.498	0.695*	1	
	Significance test-*P* value (bilateral)	0.312	0.671	0.745	0.143	0.026		
Cd	Correlation coefficient (Pearson)	0.847**	0.641*	0.835**	0.634*	−0.116	0.169	1
	Significance test-*P* value (bilateral)	0.002	0.046	0.003	0.049	0.750	0.642	
Cr	Correlation coefficient (Pearson)	1						
	Significance test-*P* value (bilateral)							
Cu	Correlation coefficient (Pearson)	0.484	1					
	Significance test-*P* value (bilateral)	0.131						
Zn	Correlation coefficient (Pearson)	0.742**	0.775**	1				
	Significance test-*P* value (bilateral)	0.009	0.005					
As	Correlation coefficient (Pearson)	0.166	0.642*	0.416	1			
	Significance test-*P* value (bilateral)	0.626	0.033	0.203				
Hg	Correlation coefficient (Pearson)	−0.423	−0.739**	−0.587	−0.691*	1		
	Significance test-*P* value (bilateral)	0.195	0.009	0.058	0.018			
Pb	Correlation coefficient (Pearson)	0.526	0.093	0.480	−0.518	0.116	1	
	Significance test-*P* value (bilateral)	0.096	0.785	0.135	0.103	0.735		
Cd	Correlation coefficient (Pearson)	0.434	0.821**	0.849**	0.593	−0.634*	0.098	1

	Significance test-*P* value (bilateral)	0.182	0.002	0.001	0.054	0.036	0.774	
Cr	Correlation coefficient (Pearson)	1						
	Significance test-*P* value (bilateral)							
Cu	Correlation coefficient (Pearson)	0.632	1					
	Significance test-*P* value (bilateral)	0.068						
Zn	Correlation coefficient (Pearson)	0.893**	.835**	1				
	Significance test-*P* value (bilateral)	0.001	0.005					
As	Correlation coefficient (Pearson)	0.284	0.879**	0.556	1			
	Significance test-*P* value (bilateral)	0.459	0.002	0.12				
Hg	Correlation coefficient (Pearson)	0.116	0.26	0.122	0.069	1		
	Significance test-*P* value (bilateral)	0.766	0.5	0.755	0.86			
Pb	Correlation coefficient (Pearson)	0.606	0.278	0.650	0.021	−0.073	1	
	Significance test-*P* value (bilateral)	0.084	0.468	0.058	0.956	0.851		
Cd	Correlation coefficient (Pearson)	0.879**	0.722*	0.976**	0.439	0.110	0.719*	1
	Significance test-*P* value (bilateral)	0.002	0.028	0.000	0.238	0.779	0.029	

Note: **correlation is significant at the 0.01 level (2-tailed);*correlation is significant at the 0.05 level (2-tailed).
